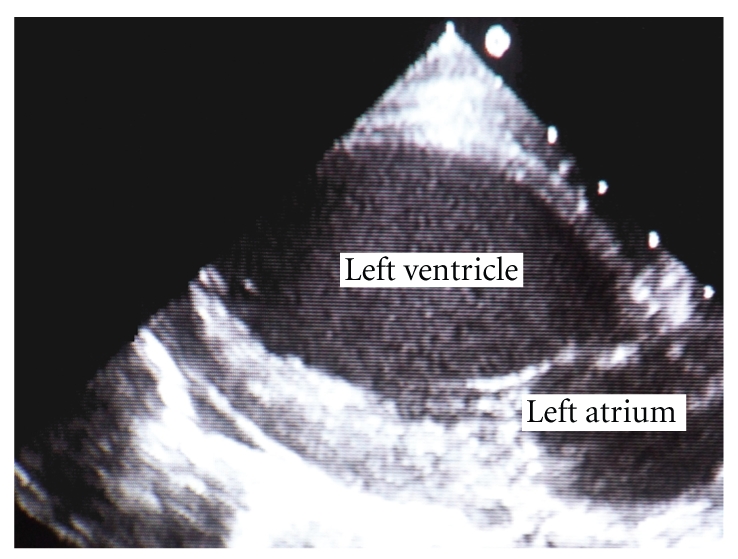# Medium-Chain Acyl-CoA Dehydrogenase Deficiency in an Infant with Dilated Cardiomyopathy

**DOI:** 10.4061/2009/281389

**Published:** 2009-12-16

**Authors:** Marcello Marcì, Patrizia Ajovalasit

**Affiliations:** ^1^Department of Cardiology, Azienda Ospedaliera “Villa Sofia & CTO”, 90149 Palermo, Italy; ^2^Department of Pediatric Cardiac Surgery, ARNAS Ospedale Civico, 90100 Palermo, Italy

## Abstract

We report about an infant affected by dilated cardiomyopathy (CMP) in whom metabolic investigations evidenced medium-chain-acyl-CoA dehydrogenase deficiency (MCADD), that is one of three types of inherited disorders of mitochondrial fatty-acid *β*-oxidation. Long-chain and very long-chain 3-hydroxyacyl-coenzyme A dehydrogenase deficits are recognized as responsible of hypertrophic or, less frequently, dilated cardiomyopathy (CMP) in childhood. Otherwise, to our knowledge, no case of MCADD associated to dilated CMP has been reported in literature.

## 1. Case Report

**Figure 1:**
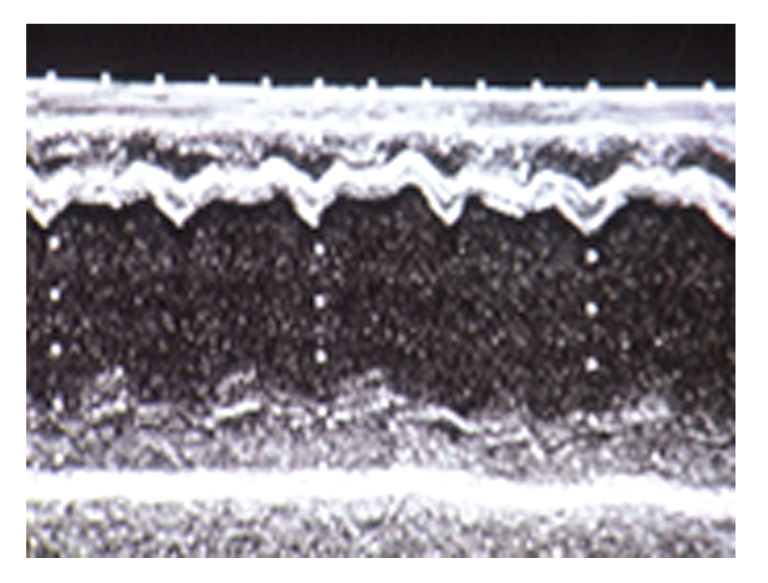


A 2-month-old female infant with severe dilated CMP, put on medication with digitalis and diuretics, was referred to our Institution to undergo heart transplant. Her twin sister died suddenly in the first few days of life, and a clear cause of death was not demonstrated. Initial physical examination revealed growth retardation, due to failure to thrive, hypotonia, S3 gallop, tachypnea, and liver enlargement. The echocardiogram showed severe left ventricular dilatation (end diastolic diameter = 45 mm) with global hypokinesis (Figure 1); furthermore an ejection fraction of 20% was noted (Figure 2). 

In addition thoracic radiograph evidenced severe cardiomegaly. No significant ventricular arrhythmias were detected. Heart failure required medical treatment with diuretics and digitalis. 

Detection of elevated urinary dicarboxylic acids prompted biochemical studies of fatty acid metabolism, which disclosed a normal carnitine level (24 umol/L). Moreover the serum acylcarnitine profile showed elevated levels of C6, C8, C10, and C10.1, that were consistent with diagnosis of MCADD. 

After initial treatment with intravenous glucose, the patient was fed with a low-fat diet and carnitine supplementation. Over the next six weeks this treatment led to a dramatic clinical improvement, and she became more active and gained 400 g. Repeated echocardiogram indicated progressive enhancement of ejection fraction to 35%; in addition left ventricular end diastolic diameter reduced to 40 mm. The patient was discharged home on 90th day. 

## 2. Discussion

Despite advances in drug therapy dilated CMP remains one of the leading cause of death and heart transplant in childhood. 

Its incidence in infancy is estimated in about 4.4 cases per 100 000 per year, with a high mortality: 50%–60% at two years. Moreover about one third of patients dies or undergoes heart transplant in the first year [1, 2]. Although the most of cases (66%) are defined “idiopathic”, some CMP may have an identifiable cause [1–4]. Identification of underlying cause of CMP in children may improve outcome after specific treatment [1, 3, 4]. Positive family history of heart failure, sudden death, or genetic syndrome is usually predictive of a probable cause for CMP [4]. 

With the exception of medium chain CoA acyl dehydrogenase deficiency, inborn disorders of fatty-acid *β*-oxygenation, have been identified as possible causes of CMP in infancy [2]. Alteration of mitochondrial beta-oxidation could be responsible of about 15% of CMP [5]. Congestive heart failure is determined by reduced energetic production due to defect of fatty-acids metabolism that reactivates the less effective foetal glycolytic pathway [6, 7]. Furthermore accumulation of nonesterified fatty acids has toxic effects on ionic channel of cardiac myocytes [5] that may trigger ventricular arrhythmias even fatal [5, 8, 9]. 

Fatty-acid *β*-oxygenation disorders are determined by an autosomal recessively inherited deficiency of a single enzyme in the mitochondrial metabolism [4, 6]. The *β*-oxidation shortens progressively very-long-chain acyl-CoA through a four-step enzymatic process: carnitine cycle, the beta-oxidation cycle, the electron transfer pathway, and the synthesis of ketone bodies. Firstly the carnitine cycle allows the long chain fatty acids to get into the mitochondria; otherwise short- and medium-chain fatty acids may enter directly. Successively the acyl-CoA, produced by the beta-oxidation cycle, can be utilized in the Kreb's cycle as well as in the production of ketone bodies. 

The metabolic disorder can involve any of the four different acyl-CoA dehydrogenases, which are of primary importance for beta-oxidation cycle. Clinical manifestations of fatty-acid *β*-oxygenation deficiencies are usually precipitated by fasting, such as infectious disease, and vary widely according to the specific enzimatic defect [2, 10–12]. MCADD is the most common error in mitochondrial beta-oxidation [13], with a prevalence of 1 case per 15 000 in USA [8] and about 4.5 cases/100 000 live births in England [14]. Its clinical consequences are variable; patients usually manifest before two years of age with life-threatening hypoketotic hypoglycaemia that can progress to coma and death [2, 8, 10]. 

 Clinical presentation of MCADD may be dramatic, even in neonatal age [8, 14], with lethargy, hypoglycaemia, acidosis, and hepatomegaly. Although ventricular tachyarrhythmias and cardiac arrest may occur, no abnormalities of myocardial function have been revealed in these patients. Mortality rate of MCADD ranges from 20% to 25% in undiagnosed patients [13]. Because prompt diagnosis and causal therapy may considerably improve outcome, diagnosis should be considered in neonates and infants with “idiopathic” CMP or life-threatening ventricular arrhythmias [5, 8], especially if family history is suggestive of unexplained sudden infant death. Treatment of fatty-acid oxidation defects includes avoiding fasting and replacement of dietary long chain by low dose of medium or short-chain fatty-acids, providing most of the calories intake as carbohydrates to reduce lipolysis, whereas L-carnitine supplementation is controversial [11]. 

The case described by us is unusual because a comprehensive review of literature indicates that MCADD has never been reported in association with dilated or hypertrophic CMP. 

## Figures and Tables

**Figure 2:**